# Reconstructing the historical expansion of industrial swine production from Landsat imagery

**DOI:** 10.1038/s41598-022-05789-5

**Published:** 2022-02-02

**Authors:** Lise R. Montefiore, Natalie G. Nelson, Amanda Dean, Mahmoud Sharara

**Affiliations:** 1 Biological and Agricultural Engineering, North Carolina State University, Campus Box 7625, Raleigh, NC 27695 USA; 2 Center for Geospatial Analytics, North Carolina State University, Raleigh, NC 27695 USA

**Keywords:** Agroecology, Computer science

## Abstract

In the USA, historical data on the period over which industrial swine farms have operated are usually only available at the county scale and released every 5 years via the USDA Census of Agriculture, leaving the history of the swine industry and its potential legacy effects on the environment poorly understood. We developed a changepoint-based workflow that recreates the construction timelines of swine farms, specifically by identifying the construction years of swine manure lagoons from historical Landsat 5 imagery for the period of 1984 to 2012. The study focused on the Coastal Plain of North Carolina, a major pork-producing state in the USA. The algorithm successfully predicted the year of swine waste lagoon construction (+ /− 1 year) with an accuracy of approximately 94% when applied to the study area. By estimating the year of construction of 3405 swine waste lagoons in NC, we increased the resolution of available information on the expansion of swine production from the county scale to spatially-explicit locations. We further analyzed how the locations of swine waste lagoons changed in proximity to water resources over time, and found a significant increase in swine waste lagoon distances to the nearest water feature across the period of record.

## Introduction

Since the mid-twentieth century, North American farms have undergone massive industrialization and transformation. Rather than raising animals on open land, such as pasture, the food animal production industry began to transition to the use of concentrated animal feeding operations (CAFOs). The CAFO model was initially pioneered by the poultry industry during the 1950s and later adopted by swine producers in the 1970–1980s^1^. Today, swine are primarily raised in CAFOs, with each CAFO raising thousands of pigs and generating large volumes of manure. Swine waste management strategies vary regionally, with one common approach relying on the use of single-stage or multi-stage anaerobic lagoons to store and treat the manure. Microbial communities in the lagoon decompose the manure organic matter, reducing its chemical oxygen demand (COD) and odor potential, while gas exchange at the lagoon surface facilitates the release of decomposition gases, CO_2_, CH_4_, NH_3_ and H_2_S. The supernatant, dilute portion of stored manure is used to flush manure from barns into storage lagoons, while the lagoon-stored manure is managed by using the liquid waste slurry as nutrient source to non-food crops in “sprayfields” adjacent to the lagoons. The lagoon-based management strategy is also used in dairy and cattle production systems.

The growth of the animal production sector is often dictated by distances to processing plants, feed mills and transportation routes to minimize transportation cost. This has led to the emergence of densely clustered CAFOs over relatively small geographic areas, resulting in massive amounts of waste being generated, stored, and land applied in “hotspots”' across the landscape^2^. In the USA, three states (i.e., Iowa, Minnesota, North Carolina) produce over 50% of the hogs produced nationally^3^. Within these states, swine production is clustered in specific areas, including the Iowa-Minnesota border and the North Carolina Coastal Plain.

In areas with dense swine production, the open-air lagoon waste management strategy is attributed to several adverse effects on surrounding ecosystems and communities, particularly in the form of air and water quality impacts^4–6^. Manure applied to adjacent fields is believed to act as critical inputs of nitrogen (N), phosphorus (P) and emerging chemical and microbiological contaminants, such as pharmaceuticals and antibiotic-resistant pathogens, to groundwater and surface waters^7–10^. Under the USA Clean Water Act, CAFOs that either discharge pollutants from point sources to national waters or use a liquid waste disposal system are required to apply for a permit through the U.S. Environmental Protection Agency (USEPA) National Pollutant Discharge Elimination System (NPDES)^11^. The locations of individual swine CAFOs can be identified through USEPA NPDES permit records. In several states where the authority to oversee implementation of the Clean Water Act has been delegated to state agencies, local departments of environmental quality oversee CAFO regulations. However, the EPA estimated that over 60% of CAFOs do not hold permits^12^. Beyond the USEPA NPDES permit records, no accurate databases exist with the locations of CAFOs^13^. In the USA, the most spatially comprehensive documentation of animal production is through the U.S. Department of Agriculture’s (USDA) Census of Agriculture, which reports the number of animals housed and processed in most counties across the USA every five years. Yet, these data are only available at the county-scale, leaving gaps in our understanding of exact CAFO locations. Without knowledge on the locations of CAFOs, their collective effects on the surrounding environment are challenging to estimate given that administrative boundaries (i.e., county boundaries) do not always align with physiographic or environmentally-relevant areas.

Several environmental and public interest groups have attempted to retrieve the coordinates of CAFOs by manually labeling CAFOs through satellite or drone imagery^14^. Other scholars have developed algorithms to automatically detect CAFOs through recent and high-resolution image satellites^14^. These studies and current permit records provide critically needed geospatial information about the CAFO industry, but the history of these operations remains largely undocumented and underexplored. Even in areas where the current locations of CAFOs are known, data on the time period over which they have operated are unavailable, leaving the history of the industry and its potential legacy effects on the environment poorly understood. Historical data are needed to complement existing datasets and understand how changes in the agriculture landscape may be related to long-term consequences on natural resources and adjacent communities.

Although satellite imagery has been used to identify the present-day locations of CAFOs, satellite remote sensing also offers opportunities to detect historical conversion of land to CAFOs. The repeated acquisition of time-ordered satellite images allows for land-use characteristics to be mapped at multiple spatial and temporal scales^15,16^. Land-use change is commonly assessed from remotely sensed data by detecting changes in radiance values^17^. Methods for detecting shifts in radiance, and land-use change by extension, include image rationing, vegetation index differencing, principal component analysis, and change vector analysis^17^. Further, over the last few decades, changepoint detection approaches have been developed to identify abrupt or structural change in the distributional properties of data (e.g., mean, variance)^18,19^. At Most One Change^20^, Binary Segmentation^21^, Bayesian Online Change Point Detection^22^, and Pruned Exact Linear Time^23^ are popular changepoint detection methods that have applications in satellite remote sensing.

This study aims to answer the question: How long have existing swine CAFOs been in operation? We specifically focus on swine CAFO waste lagoons, instead of barns, when analyzing CAFOs; lagoons serve as concentrated sources of nutrients at the farm-scale (i.e., one lagoon can receive inputs from multiple barns), making lagoons ideal features to analyze. To answer the outlined research questions, the study was structured with the following objectives: (1) Develop a generalizable and scalable changepoint-based algorithm for recreating the construction timelines of swine waste lagoons from historical satellite imagery and (2) Apply the algorithm to generate a spatially-explicit dataset of swine waste lagoon construction years. Additionally, we quantified the distances of swine waste lagoons to nearby waterbodies over time as a representative example of how the data can be used for exploratory analyses of relationships between long-term CAFO construction trends and the environment.

## Methods

### Study area

**Figure 1:**
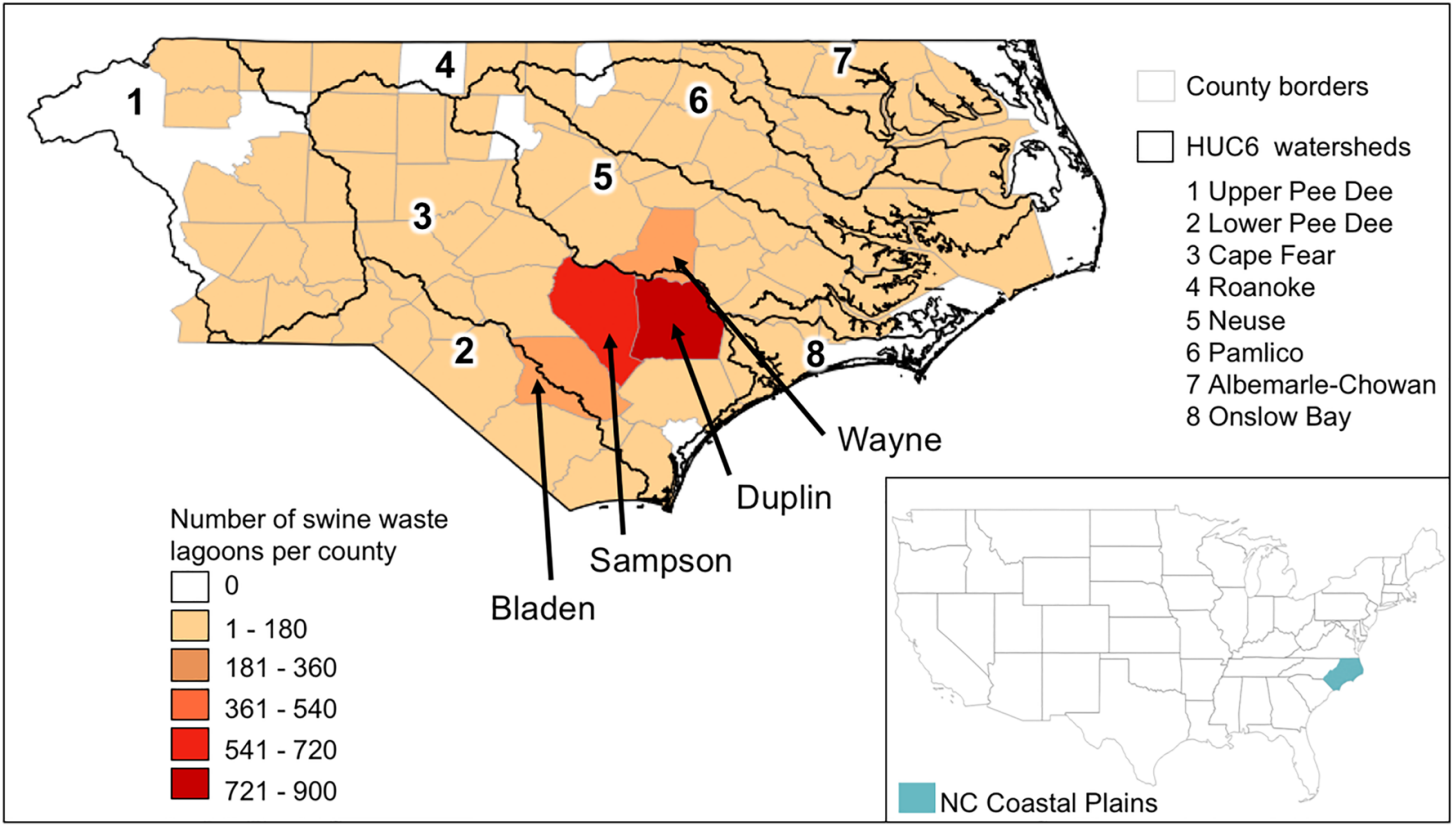
Study area, composed of the North Carolina (NC) Coastal Plain, USA. County boundaries are shown as gray lines, with the fill color corresponding to swine lagoon density (i.e., number of swine waste lagoons within the county). Watershed boundaries (HUC6) are shown with black lines, and labeled with numbers. The inset map in the bottom right shows the location of the study area (NC Coastal Plain) in the USA shaded in blue. This figure was produced using QGIS version QGIS 3.18.3 (https://www.qgis.org/).

We focused on North Carolina (NC), a major pork-producing state in the USA, as a representative study system. NC is located along the Atlantic Ocean and covers an area of 139,390 km^2^. Swine production is primarily concentrated in the eastern center of the Coastal Plain of North Carolina, especially in Duplin and Sampson Counties (Fig. 1). Eight major river basins drain the Coastal Plain of NC, and wetlands are common throughout the low-lying region.

According to the USDA county-scale animal inventory, NC’s domesticated swine population jumped from 2.54 million in 1987 to more than 9.62 million in 1997^24,25^. In 2017, over 8.89 million hogs were produced in NC and sold domestically and internationally^3^. The growth of industrialized swine production also led to the construction of over 3,000 swine waste lagoons^26^, which collectively store several million tons of manure produced annually. In NC, swine CAFOs and associated waste lagoons were largely constructed prior to August 27th 1997, at which time a moratorium was placed on the construction and expansion of swine farms in an effort to control the explosive growth of the industry^27^. The moratorium was made permanent in 2007 for farms that use swine waste lagoons as the primary form of waste treatment. Presently, swine CAFOs that use waste lagoons are permitted and covered by the NC Swine Waste Management System General Permit and their locations can be retrieved from the North Carolina Department of Environmental Quality (NCDEQ). Although the post-1997 (i.e., present-day) locations of swine CAFOs are known, the coordinates correspond to property locations, and not the locations of lagoons within CAFO properties. Moreover, dates when individual lagoons were constructed are not included in NCDEQ permit records. Consequently, the spatiotemporal expansion of the swine industry prior to the moratorium is only documented through county-scale data from the USDA Census of Agriculture, and not through spatially-explicit records.

### Characterizing swine waste lagoon construction using satellite remote sensing

**Figure 2:**
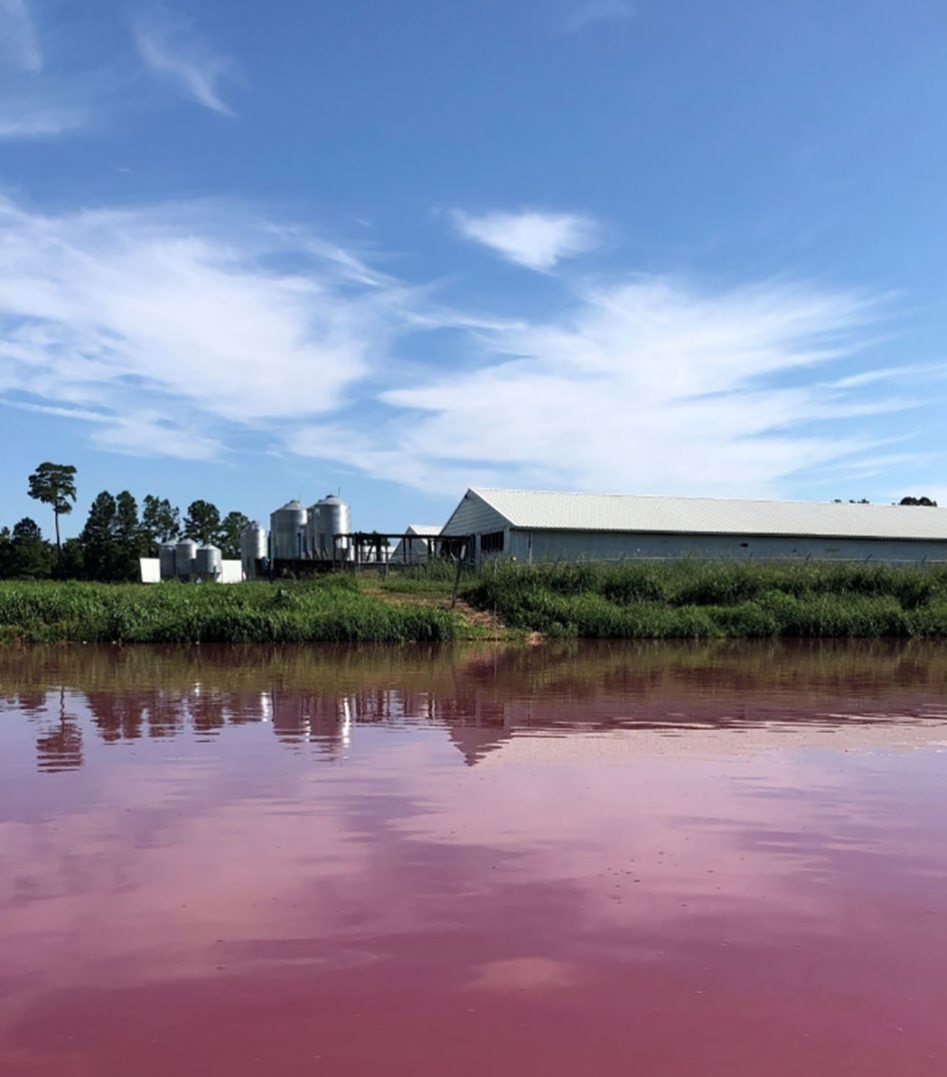
Swine operation and lagoon in Eastern North Carolina (July 2019). Photograph by Mahmoud Sharara.

To identify the year in which each swine waste lagoon in NC was constructed, multi-temporal Landsat 5 imagery of NC was analyzed. Because the locations from permit records do not correspond to specifically the locations of individual waste lagoons, only the farms, the exact swine waste lagoon site coordinates were retrieved by inspecting high resolution and recent satellite imagery (2018) in Google Earth Pro. Due to waste lagoons sometimes having overlapping spectral features with some waterbodies (i.e. those with high organic matter), a supervised classification approach for automatically detecting lagoon locations was not feasible. However, swine waste lagoons are visually characterized by rectangular shapes and brown/pink color, and are located near barns (Fig. 2). Therefore, they can be easily spotted by the human eye on high-resolution images provided by Google Earth Pro. Thus, spatial data points of swine waste lagoons were manually tabulated and a total of 3,405 waste lagoons were identified across the study area. Surface reflectance at the locations of the 3,405 existing lagoons were analyzed over the Landsat 5 Surface Reflectance Tier 1 collection through Google Earth Engine^28^ from 1984 to 2012. Atmospherically corrected surface reflectance from the Landsat 5 ETM sensor was used for this study due to its temporal coverage (1984–2012), which coincides with the growth period of the swine CAFO industry (1980s–1997), and the high spatial and temporal resolution of the images (30 m, one image every 16 days). The average swine waste lagoon surface area was large enough to be resolved by the resolution of Landsat 5 images (average and median surface area of approximately 6,600 and 5,200 m^2^, respectively, in the validation set described below). Time-ordered images of Landsat 5 were filtered to keep only images with cloud cover less than 5%. Further, all images met the geometric and radiometric quality requirements and only images with a quality of 9 (i.e., best quality) were used. A total of 959 Landsat 5 images were used for the study area. An average of 123 Landsat 5 images were available for the period 1984 to 2012 and at least one image was available per year for each waste lagoon.

The Near Infrared (NIR) band of Landsat 5, also called band 4 (B4; 0.76–0.90 μm) was analyzed at each swine waste lagoon location. While water strongly absorbs light in the B4 wavelength range, land does not. Thus, we expected to observe a dramatic B4 value change when a pixel transitioned from a non-watery (i.e., crop, forest, pasture) to watery feature (i.e., swine waste lagoon). In addition to B4, the Normalized Difference Water Index (NDWI) was tested for its ability to capture conversion from non-watery to watery pixels. NDWI was also found suitable for the analysis, but B4 was selected because the computation was more efficient in Google Earth Engine than for the NDWI.

B4 time series were extracted from the Landsat 5 images. Time-points with surface reflectance values greater than 10,000 caused by bright features (e.g., clouds) were removed from the analysis, and linear filtering was applied to the B4 reflectance time series methods to reduce noise in the data. Data cleaning was performed in R^29^.

### Validation data

**Figure 3:**
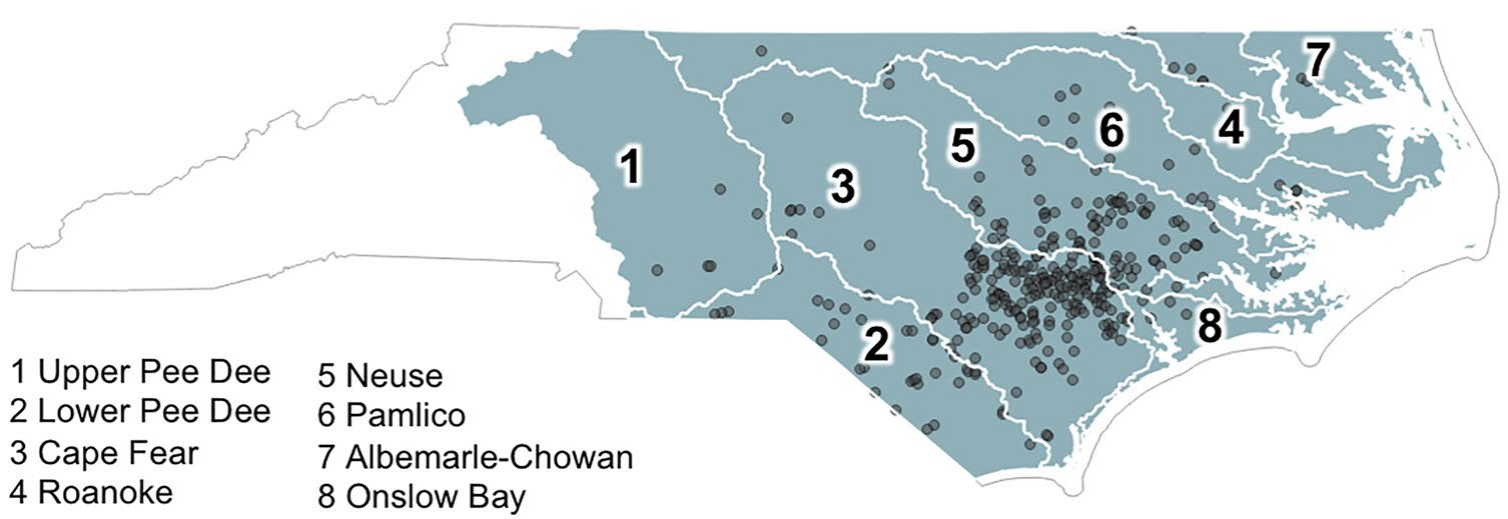
Swine waste lagoon sites (n = 340) randomly selected to compose the validation set. The sample is spread across the eight HUC6 watersheds in the study area (watershed boundaries shown as white lines and labelled with numbers). This figure was produced using QGIS version QGIS 3.18.3 (https://www.qgis.org/).

Ten percent (n = 340) of the total number of swine waste lagoons (n = 3,405) were randomly chosen and further analyzed to produce a validation dataset of their respective construction years (Fig. 3). Landsat 5 and high-resolution historical images through Google Earth Pro were used to visually verify the year of construction for each of the 340 swine waste lagoons in the validation set.

### Estimating swine waste lagoon construction years using changepoint detection

A changepoint detection analysis was run on each of the B4 reflectance time series (n = 3,405) to automatically detect abrupt changes in reflectance, which were assumed to occur due to lagoon construction. The years in which changepoints occurred were then tabulated as estimates of the construction year for each lagoon across the study area. To perform the changepoint analysis, the *changepoint* R package was used^30^.

Specifically, the Binary Segmentation (BinSeg) changepoint detection algorithm was tested. The BinSeg algorithm can detect changes in the mean, variance, or both mean and variance across a time series and under different assumptions on the distribution of the data (e.g., exponential, normal). Once a changepoint is detected, the time series is split into two distinct homogeneous segments: S1 (i.e., the series prior to the changepoint) and S2 (i.e., the series after the changepoint). The BinSeg algorithm was evaluated under assumptions of a normal distribution (BinSeg-Normal). We also evaluated two metrics with which to assess changepoints: mean, and the combination of mean and variance (MeanVar). Performance of the changepoint algorithms BinSeg-Normal-Mean and BinSeg-Normal-MeanVar were evaluated using percentage error by comparing model outputs with observations (i.e., number of misclassifications relative to total sites) in the validation set (n = 340) (Fig. 3). Further, lagoons for which the algorithms did not correctly estimate the construction year (n = 19) were further analyzed to understand potential sources of error by visually inspecting historical Google Earth Pro images.

We expected to observe an abrupt and distinct jump in the B4 time series from higher reflectance values (i.e., vegetation pixel) to lower reflectance values (i.e., swine waste lagoon pixel) coincident with swine waste lagoon construction. We assumed that this critical land-use transition overcame other external and internal systematic factors (e.g., organic matter content, bacteria) and corresponded to the greatest shift in the mean and MeanVar of the B4 reflectance time series. We also assumed that the pixel only underwent one major land use conversion (i.e., to waste lagoon) given that waste lagoons have largely remained permanent on the land surface upon construction. Therefore, the algorithms were parameterized to detect only one changepoint.

In order to successfully identify changepoints, satellite images had to be acquired before and after the year of swine waste lagoon construction. Therefore, because Landsat 5 images were available from 1984 to 2012, the years of construction could only be detected accurately for swine waste lagoons built from 1986 to 2010.

### Spatiotemporal distribution of swine waste lagoons in relation to water resources

To evaluate how the locations of swine waste lagoons varied in relation to water resources over time, the nearest distance between swine waste lagoons and waterbodies was computed over the period of record. Watershed boundaries were downloaded from the National Watershed Boundary dataset^31^ specifically for the U.S. Geological Survey (USGS) Six-Digit Hydrologic Units (HUC6) and Twelve-Digit Hydrologic Units (HUC12). Furthermore, the National Hydrography Dataset (NHD)^32^ was used to map waterways. The NHD was filtered to keep surface water features, specifically reservoir, canal/ditch, lake/pond, stream/river, and estuary. Nearest water features to swine waste lagoons were identified and the distances were computed through ArcGIS^33^ with the geodesic method. Spatial metrics of swine waste lagoons in relation to water resources were extracted and summarized via ArcGIS and visualized in R. The Mann–Kendall statistical test^34,35^ was used with a 5% alpha level to assess whether the annual median distance between the waste lagoons and the nearest waterways increased significantly over time.

## Results

### Changepoint detection method

Although most of the reflectance time series used in the BinSeg–Normal–Mean and BinSeg–Normal–MeanVar algorithms had a normal distribution, several lagoons had distributions that were skewed or did not follow a normal distribution (Fig. S1). However, results suggested that the accuracy of the detected changepoints were not sensitive to the normality assumption or distributional characteristics.

The BinSeg-Normal-Mean algorithm had the highest performance (81% of the 340 validation sites) in detecting the correct year of swine waste lagoon construction, followed by BinSeg-Normal-MeanVar (77%). The two algorithms did not detect the same year of construction for 19 waste lagoons; of these 19, the BinSeg-Normal-Mean detected the correct year for 84% of them, while the BinSeg-Normal-MeanVar detected the correct year for only 16%. Therefore, the BinSeg-Normal-MeanVar algorithm was abandoned given it did not provide additional useful information relative to the BinSeg-Normal-Mean algorithm.

Despite good performance, the BinSeg-Normal-Mean algorithm consistently detected a changepoint during the period of record for all sites included in the 10% validation set (n = 340 swine waste lagoons). However, 58 of the 340 swine waste lagoons were constructed prior to 1986, before the period of record suitable for detecting an accurate changepoint. Changepoints before 1986 either (1) detected the correct construction year, or (2) incorrectly detected a changepoint due to artifact signals identified on the images taken in 1984, probably associated with the initial satellite commissioning. In the latter circumstance, if the algorithm detected a changepoint due to this signal, it meant that no land-use change was detected after 1986. Therefore, these waste lagoons were estimated as having been constructed before 1986. In some conditions, when a large number of images was available for the year 1985 and 1986, the algorithm was able to detect the changepoint occurring for the years 1985 or 1986. Further, the BinSeg-Normal-Mean algorithm detected a false year of construction for swine waste lagoons for which the mean of the segment after the changepoint (S2) had a greater average than the segment before the changepoint (S1).

**Figure 4:**
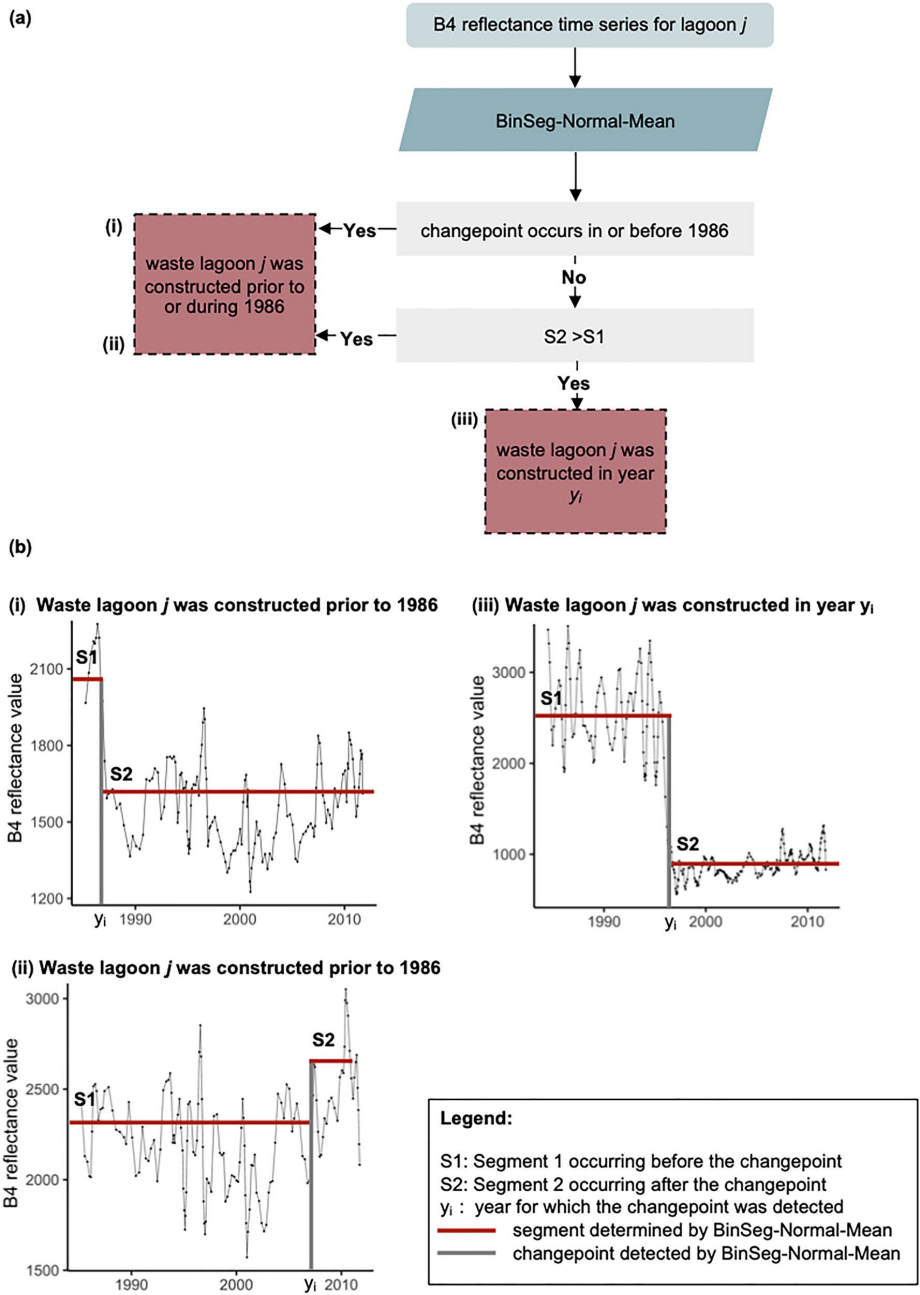
Changepoint detection algorithm for determining the year of construction of swine waste lagoons. Panel (**a**) summarizes the algorithm workflow, while panel (**b**) illustrates specific examples corresponding to each step (i–iii) in the workflow.

To increase algorithm performance, we developed a workflow to address some of the aforementioned caveats (Fig. 4). In this workflow, the BinSeg-Normal-Mean algorithm is applied to a B4 reflectance time series at location j. If the BinSeg-Normal-Mean changepoint is identified for a time in or prior to 1986 (Fig. 4a,i,b,i) we assume that the lagoon was constructed in or prior to 1986. Similarly, a lagoon is assumed to be constructed in or prior to 1986 if a BinSeg-Normal-Mean changepoint is identified after 1986 and the mean of S2 is greater than the mean of S1 (Fig. 4a,ii,b,ii). If a changepoint occurred after 1986 and the mean of S1 was greater than S2, then the changepoint was estimated as having occurred between 1987 and 2010 (Fig. 4a,iii,b,iii).

**Figure 5:**
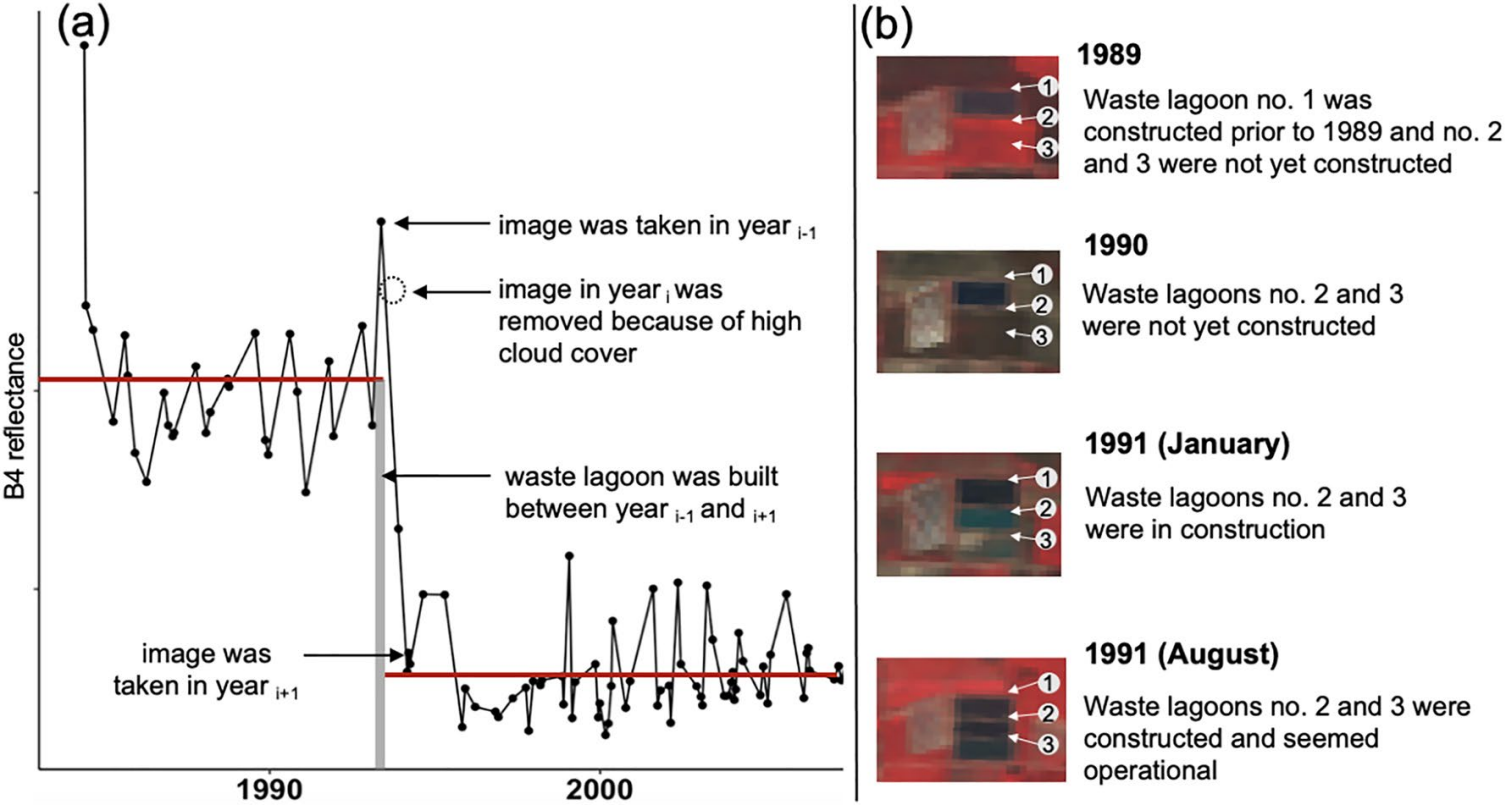
Examples of limitations to the changepoint detection algorithm. In some cases, an insufficient number of high-quality Landsat 5 images were available to capture the year of construction of an individual swine waste lagoon (**a**), resulting in errors of + /− 1 year. In other cases, the changepoint algorithms detected the start of the construction of the swine waste lagoon but the swine waste lagoon was not fully operational until later years due to prolonged construction timelines (**b**).

The performance of the workflow was evaluated using the validation set composed of 10% of the total number of swine waste lagoons (n = 340). With the new approach, 94% of the swine waste lagoon construction years (+ /- one year) were accurately retrieved. A tolerance of + /− 1 year was chosen to account for a lack of images in some years due to issues with image quality (e.g. high cloud cover) (e.g., Fig. 5a), or because construction spanned at least a year (e.g., Fig. 5b). The changepoint detection workflow incorrectly estimated the construction years for 19 of the 340 swine waste lagoons in the validation set; the differences between the observed and predicted years of construction of these lagoons ranged from 2 to 26 years with a median of 8 years.

By visually inspecting historical Google Earth images for each of the lagoon sites for which the model incorrectly estimated construction year, we identified that model errors were associated with swine waste lagoon expansion, pixel transitions to land-use classes other than swine waste lagoons, or issues with pixels being partly covered by clouds or incompletely covered by the lagoon (i.e., narrow and small waste lagoons that do not entirely cover a pixel).

### Estimating swine waste lagoon construction years

**Figure 6:**
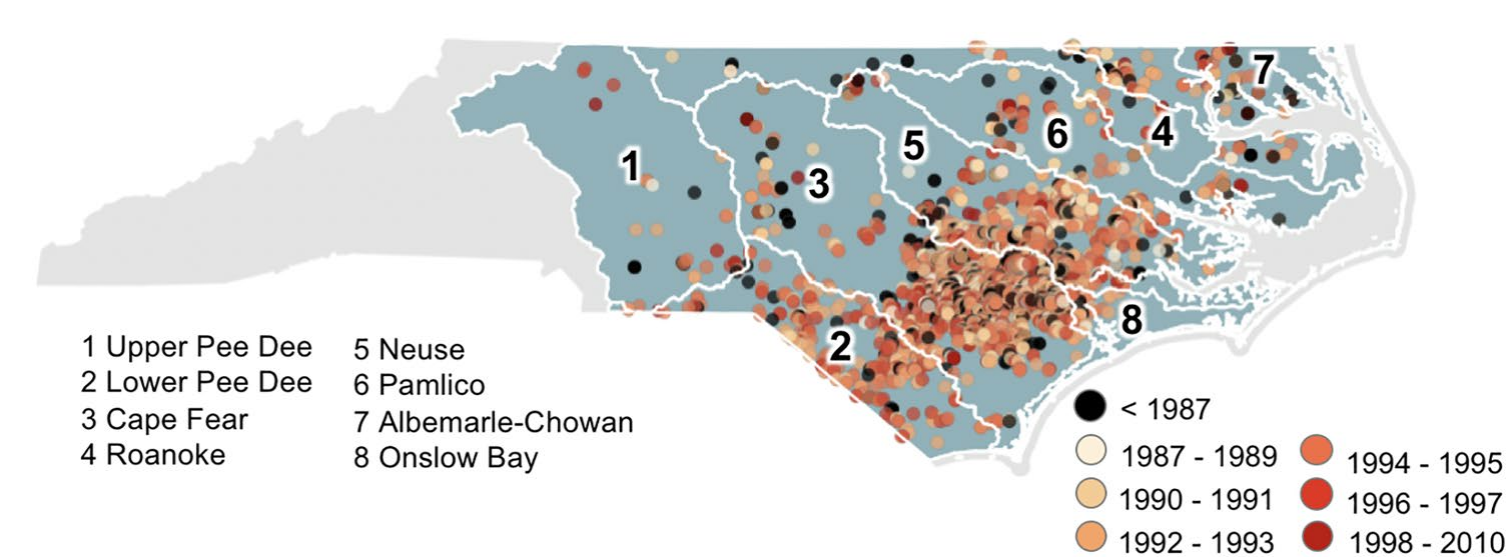
Spatiotemporal distribution of swine waste lagoon construction (+/- 1 year) across the HUC6 watersheds. This figure was produced using QGIS version QGIS 3.18.3 (https://www.qgis.org/).

Using the newly developed algorithm (Fig. 4), construction years were estimated for each swine waste lagoon in the NC Coastal Plain (Fig. 6); the years of construction for each swine waste lagoon are included in the supplementary material. Most swine waste lagoons were built in the early 90s and prior to the moratorium of 1997. More specifically, 80% of the swine waste lagoons (n = 2,736) were built between 1987 and 1997. Sixteen percent of the swine waste lagoons were constructed in or prior to 1986. A large decrease in the construction of swine waste lagoons occurred after the moratorium of 1997, with only 3.7% of swine waste lagoons being constructed after the moratorium. These results suggest that the 1997 moratorium did not completely halt the construction of lagoons, but dramatically slowed the rate of expansion.

**Figure 7:**
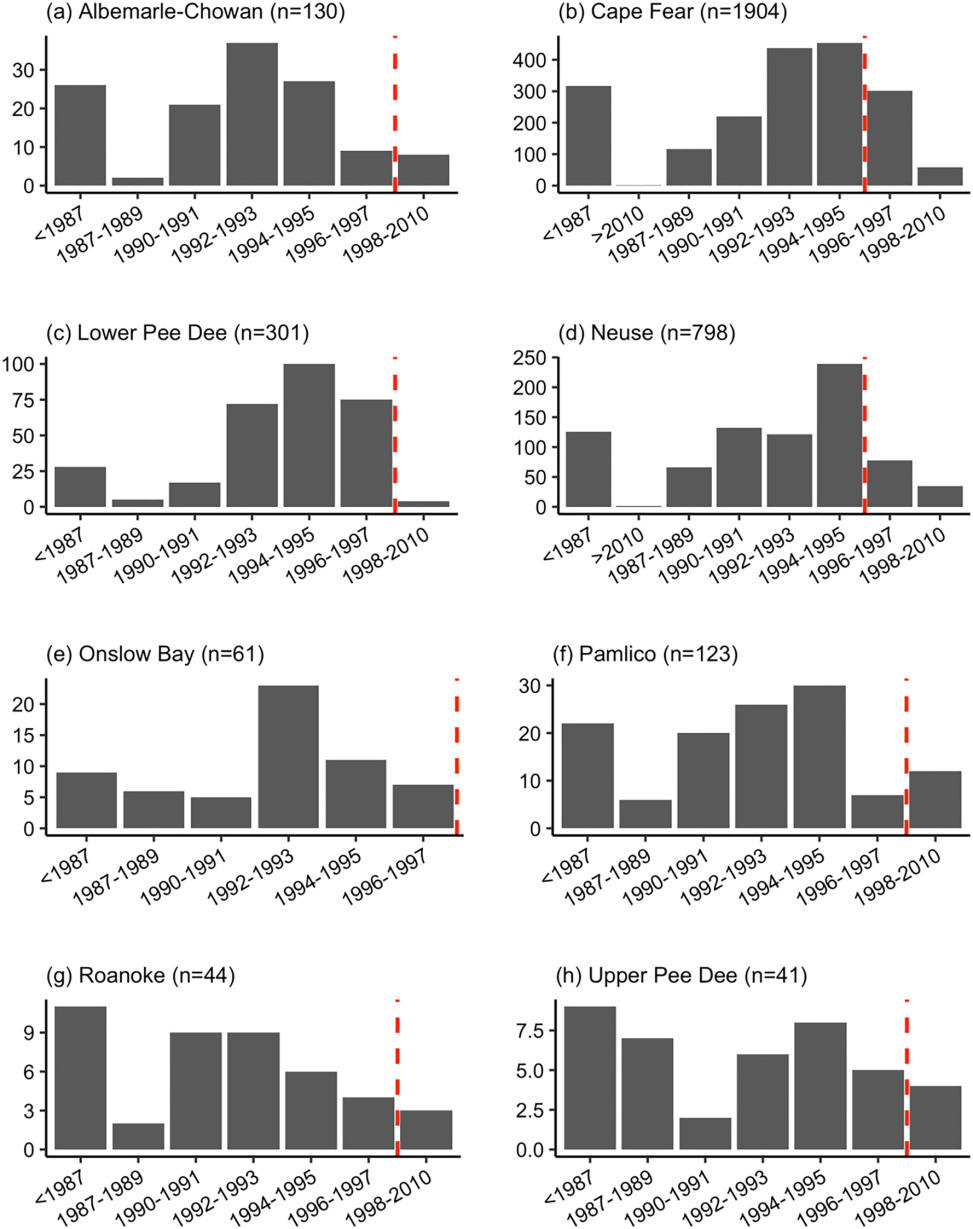
Year of construction of the swine waste lagoons (+ /− 1 year) for the HUC6 watersheds. The y-axis scales are unequal between the plots to improve readability. The dashed red lines correspond to the establishment of the moratorium in 1997.

With regards to hydrological boundaries (Fig. 7a–h), the Cape Fear River watershed had the highest number of swine waste lagoons (i.e., 56%; Fig. 7b), followed by the Neuse River (i.e., 23%; Fig. 7d), the Lower Pee Dee River (i.e., 9%; Fig. 7c) watersheds. The Albemarle-Chowan (Fig. 7a), Onslow Bay (Fig. 7e), Pamlico (Fig. 7f), Roanoke (Fig. 7g), and Upper Pee Dee (Fig. 7h) watersheds all had less than 9% of the total lagoons within the study area.

Results suggested that the Cape Fear River watershed was the center of the historical growth of the swine industry, where over 300 swine waste lagoons were built prior to 1987. The Cape Fear River watershed experienced a steady increase in the number of swine waste lagoons from 1987 to 1990, with an average of 46 swine waste lagoons being built annually. However, after 1991, the pace of swine waste lagoon construction increased dramatically with an average of 192 swine waste lagoons built annually between 1991 and 1997. The highest construction rate occurred in 1994, with 242 swine waste lagoons built. However, after the 1997 moratorium, the construction rate decreased dramatically; in 1997, 153 swine waste lagoons were constructed, and this number dropped to 23 in 1998. After 1998, the annual average number of swine waste lagoons constructed plunged to 5. Although the swine waste lagoon construction rate fell considerably after the 1997 moratorium, the decrease had already started in 1995. The same pattern was observed for the Neuse, Pamlico, Albemarle-Pamlico, and Onslow Bay watersheds.

**Figure 8:**
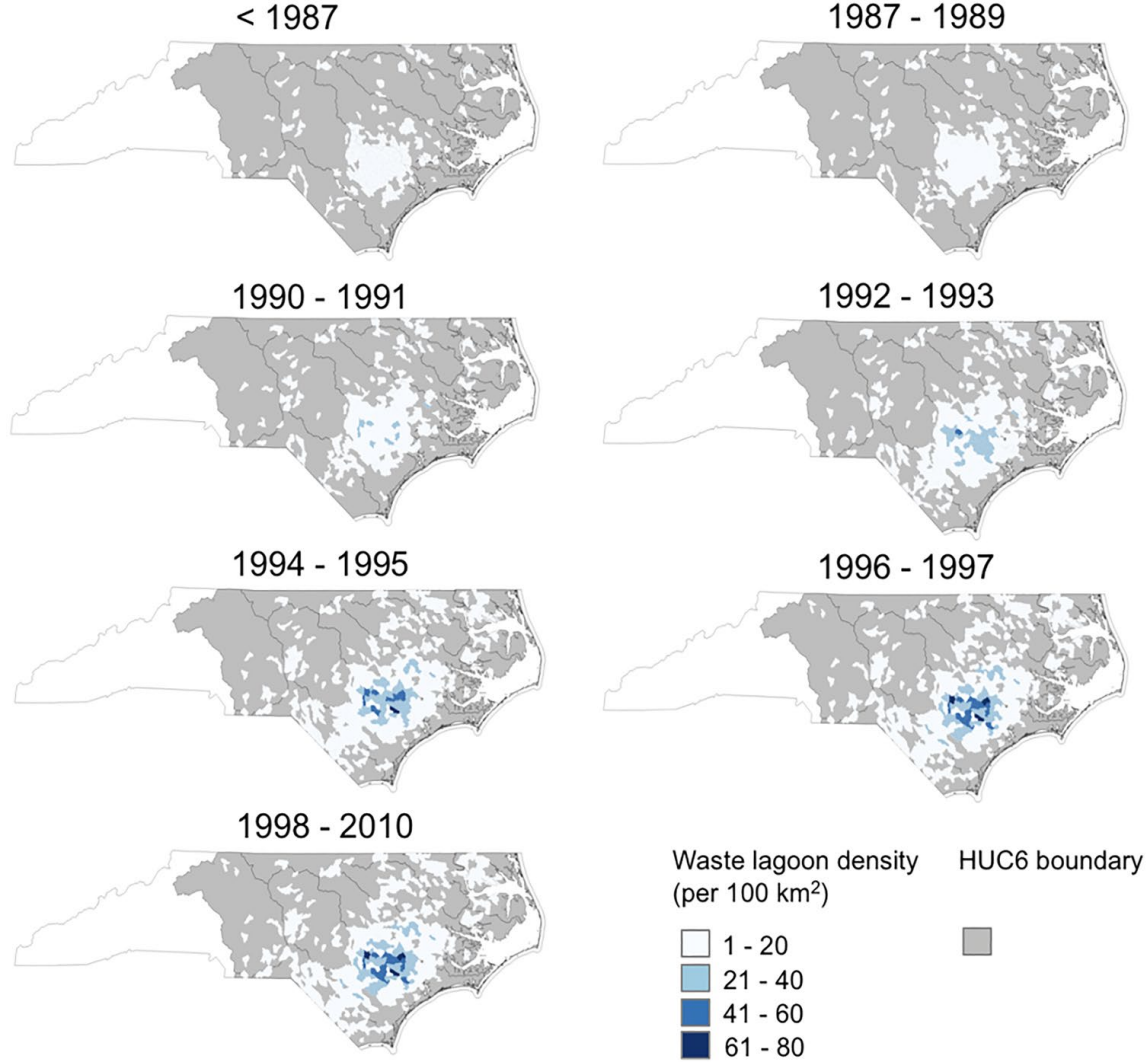
Cumulative swine waste lagoon density per 100 km^2^ reported at the HUC12 watershed scale; HUC6 watersheds shown in gray for reference. This figure was produced using QGIS version QGIS 3.18.3 (https://www.qgis.org/).

The spatiotemporal distribution of swine waste lagoons at the HUC12 watershed scale emphasized the historical clustering of the swine industry in the NC Coastal Plain. After the moratorium, swine waste lagoons were present within 436 HUC12 watersheds. However, before 1986, they were spread across only 197 HUC12 watersheds (Fig. 8). Before 1986, the density of waste lagoons was relatively low with an average of 3.38 swine waste lagoons per 100 km^2^ and a maximum of 15.13 swine waste lagoons per 100 km^2^ (i.e., Clayroot Swamp-Swift Creek watershed) (Fig. 8). In the 90s, swine waste lagoon construction expanded and continued to intensify in the region. After the moratorium of 1997, the average density of waste lagoons per HUC12 watersheds was 10 per 100 km^2^ with a maximum of 78 waste lagoons per 100 km^2^ identified in the Maxwell Creek-Stocking Head Creek basin. After 1997, 16 of 436 HUC12 watersheds had a swine waste lagoon density greater than 40 per 100 km^2^ (Fig. 8).

### Spatiotemporal distribution of swine waste lagoons in relation to water resources

**Figure 9:**
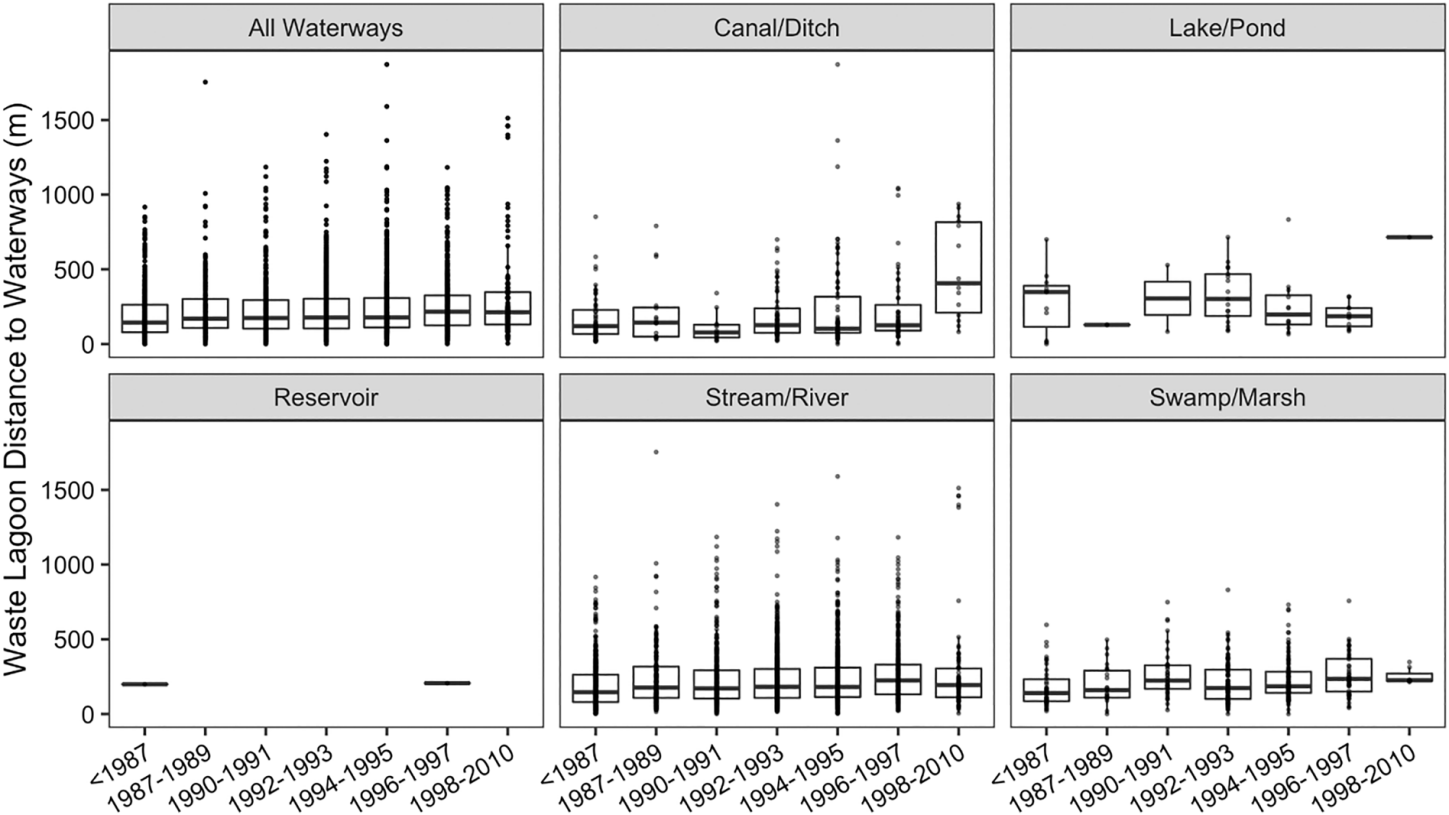
Nearest water features distance to swine waste lagoons.

Distance of swine waste lagoon sites to the nearest water feature (i.e., reservoir, canal/ditch, lake/pond, stream/river, estuary) were assessed using the NHD. The analysis revealed that over 150 swine waste lagoons were misclassified by the NHD and were documented in the NHD as lake/pond (n = 102) or swamp/marsh (n = 46). Further, we observed that some NHD water features were misclassified as other non-water features (e.g., forest, pasture), and most of these misclassifications were for polygons with an area less than 0.05 km^2^. Therefore, NHD water features with areas less than 0.05 km^2^ were removed from subsequent analyses. Distances between swine waste lagoons and waterways were computed from the NHD without features with areas less than 0.05 km^2^. The new analysis revealed that 3 swine waste lagoons remained misclassified as lake/pond (n = 1) and swamp/marsh (n = 2). Canal/Ditch, lake/pond, stream/river, and swamp/marsh were identified as the NHD features that were most commonly near swine waste lagoons (Fig. 9). Two swine waste lagoons were near a reservoir in which one was identified as a treatment-sewage pond by the NHD.

The average and median distance of all swine waste lagoons (including those built early and late in the period of record) to the nearest water features were 234 and 177 m, respectively. Further, 92% of the swine waste lagoons were less than 500 m from the nearest waterways. The Mann–Kendall results revealed a significant upward trend over time of swine waste lagoon distances to the nearest water features (alpha = 0.05, p-value = 0.01). A slight increase over time of swine waste lagoon distances to the nearest water feature is also documented in Table 1.

**Table 1:** Temporal average and median of nearest distance (m) of swine waste lagoons to water features. NA indicated that the water feature was not the closest waterway to any of the studied swine waste lagoons for the time period.

	All Waterways		Canal Ditch		Lake/Pond		Reservoir		Stream/River		Swamp/Marsh	
	mean	median	mean	median	mean	median	mean	median	mean	median	mean	median
< 1987	192	144	175	120	311	350	198	198	192	145	181	140
1987–1989	237	171	221	143	128	129	NA	NA	244	176	208	161
1990–1991	231	174	106	78	306	306	NA	NA	231	171	270	224
1992–1993	230	177	198	127	331	302	NA	NA	234	183	208	174
1994–1995	239	178	243	102	256	197	NA	NA	239	181	234	185
1996–1997	262	216	231	125	191	185	206	206	269	335	264	236
> 1998	314	213	496	407	715	715	NA	NA	283	194	253	226

## Discussion

In the present work, we developed an algorithm to reconstruct the spatiotemporal expansion of the swine CAFO industry, specifically by focusing on the construction of swine waste lagoons in North Carolina built between 1986 to 2010, from Landsat 5 satellite images. The algorithm successfully predicted the year of swine waste lagoon construction (+ /− 1 year) with accuracy of approximately 94%. By estimating the year of construction of 3,405 swine waste lagoons in NC, we increased the resolution of available information on the expansion of swine production from the county scale (i.e., via the USDA Census of Agriculture), to spatially-explicit locations. However, the algorithm performed poorly for small waste lagoons (i.e., surface area less than 900 m^2^) as they were not sufficiently resolved by the Landsat 5 images (i.e., resolution of 30 m).

Because the swine waste lagoons’ years of construction were retrieved at their exact coordinates, trends in the growth of the swine industry over time could be aggregated at different spatial scales (e.g., hydrologic, ecologic), and not only at the county scale. For example, although Duplin and Sampson Counties are already known as the center of the swine industry in NC, analysis of results from this study allowed for summaries of swine waste lagoon construction trends to be analyzed within other physiographic, instead of administrative, boundaries. Specifically, our results showed that swine waste lagoon construction in the Cape Fear River watershed, which contains Duplin and Sampson Counties, is highly concentrated: 72% of the swine waste lagoons in the Cape Fear River watershed were built on 12% (i.e., 3,031 km^2^) of the total watershed area (i.e., 23,847 km^2^). Much of the expansion in the Cape Fear River watershed occurred in 1994–1995, when 25% of all swine waste lagoons in the watershed (n = 835) were constructed. This spatial concentration of swine CAFOs raises several environmental concerns. For example, the amount of manure generated in the Cape Fear River watershed annually was estimated to equal to 41,400 and 13,630 tons of N and P, respectively^36^. These spatially clustered nutrients disturb the natural nutrient cycling of the watersheds and can lead to several long-term adverse effects on water quality^37^. Excess nutrients are subject to long transit times that can range from a few years to several decades, resulting in the accumulation of legacy nutrient stores^38,39^. By outlining the duration of time in which lagoons have been present on the landscape, data presented from this study could help to further refine estimates related to legacy nutrient dynamics in soils and groundwater, ultimately leading to improved understanding of local nutrient cycling.

Additionally, we found that the nearest waste lagoons to waterways were primarily built prior to 1986. The median distance of lagoons increased from approximately 192 m in 1986 to 314 m in 1997, indicating that lagoons progressively were built further from waterways over time, which may reflect growing understanding among the agricultural and environmental resource management communities that waste lagoon management can threaten the quality of surface waters. It is worth noting that the first lagoons constructed in NC date back to the early 1960s, with lagoons initially considered as a treatment step prior to direct discharge^40^. Research during the early 1970s showed that the treatment taking place in the lagoon insufficiently reduces nutrient and chemical oxygen demand (COD) to generate a dischargeable effluent^41^. Alternatively, this trend could also simply reflect changes in space constraints as the industry grew.

Our detailed assessment of swine waste lagoon construction timelines also revealed that the NHD, which is widely used in hydrologic and ecological studies, misclassified several swine waste lagoons as ponds or lakes. Similar errors have been reported by Martin et al. (2018)^42^ with the National Land Cover Database (NLCD). Martin et al. (2018) found that 12% of the CAFOs in North Carolina were classified as natural systems (e.g., wetlands, forests, grasslands) by the NLCD. Such misclassification can have repercussions for many studies that use the NHD for habitat assessments or water quality model parameterization. Future analyses could also retroactively correct historical data products, like prior versions of the NHD, using data generated from this study.

Although the changepoint detection algorithm effectively estimated swine waste lagoon construction years, it is not without limitations. Constructing B4 time series from which to identify changepoints requires a manual census of waste lagoons to retrieve their coordinates. This essential step demands considerable human resources and time depending on the accuracy of available data and permit records. To overcome this issue, the use of machine learning approaches (e.g., deep learning, object detections) could be used to detect the presence of CAFOs and waste lagoons on the landscape over historical image records. Previous research has successfully detected the location of CAFOs in North Carolina by applying a deep convolutional neural network to recent high-resolution satellite images^14^, though prior studies have not recreated timelines over which lagoons and CAFOs were constructed. Such computer vision algorithms could be expanded to facilitate further automation and optimization of the approach applied in the present work, though computer vision algorithms require high quality training data, which are often challenging to produce. Outputs of this study could potentially be used to create training datasets for other computer vision-based algorithms that could be applied over larger spatial scales, e.g. the entire USA. Moreover, our approach has not been tested for its ability to accurately detect the construction of non-swine waste lagoons (e.g. dairy), and further research is needed to understand the scalability of our algorithm to other animal waste systems. Additionally, the reconstructed spatiotemporal history of the swine sector in NC can provide insights into the emergence of new agricultural ventures through agent-based modeling tools as informed by spatial arrangement of transportation routes, feed mills, and processing facilities. Such insights can help inform planning in regions where animal agriculture is currently growing, such as Oklahoma, Texas, and Idaho.

## Supplementary Information

Supplementary Information 1.

Supplementary Information 2.
