# Supplementary material for: Reconstructing the historical expansion of industrial swine production from Landsat imagery
Source: Sci Rep. 2022 Feb 2;12:1736. doi: 10.1038/s41598-022-05789-5 (PMC8810989; doi:10.1038/s41598-022-05789-5)
Supplement: Supplementary file 1 — Supplementary Information 1. [file 41598_2022_5789_MOESM1_ESM.docx]

**Supplementary Material**

**Reconstructing the historical expansion of industrial swine production from Landsat imagery**

Lise R. Montefiore^1^, Natalie G. Nelson^1,2,^*, Amanda Dean^1^, Mahmoud Sharara^1^

^1^ Biological and Agricultural Engineering, North Carolina State University, Raleigh, NC, 27695

^2^ Center for Geospatial Analytics, North Carolina State University, Raleigh, NC, 27695

* Corresponding author: Campus Box 7625, Raleigh, NC 27695; [nnelson4@ncsu.edu](mailto:nnelson4@ncsu.edu)


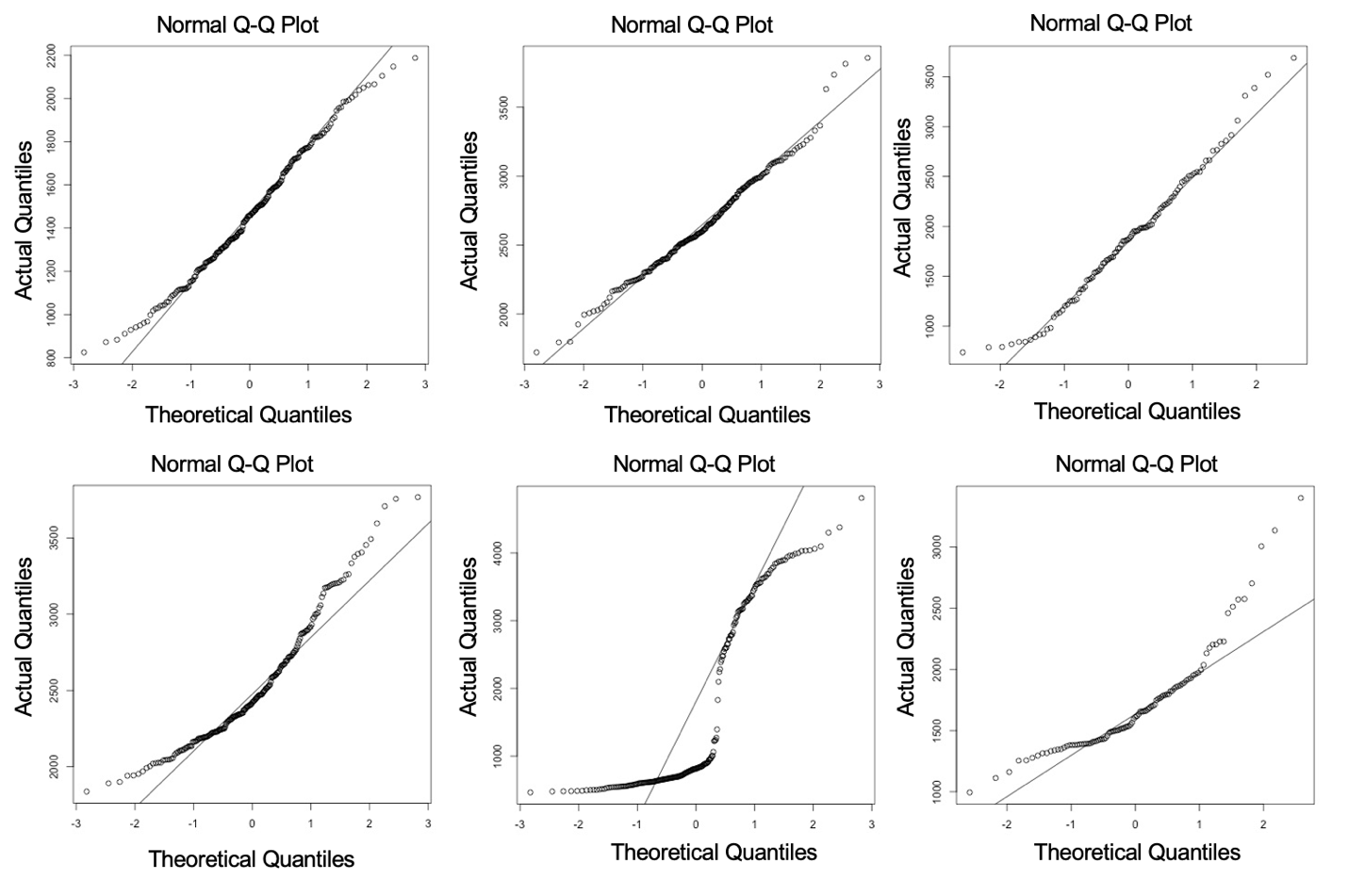


Figure S1: Normal Q-Q plots of the reflectance time series for six randomly selected waste lagoons.
